# Changes in fibrin clot properties in patients after Roux-en-Y gastric bypass surgery

**DOI:** 10.1016/j.rpth.2024.102361

**Published:** 2024-03-01

**Authors:** Kazim Abbas, Stephen J. Hierons, Nikoletta Pechlivani, Fladia Phoenix, Robin Alexander, Rhodri King, Ramzi A. Ajjan, Alan J. Stewart

**Affiliations:** 1 Renal Transplant Unit, Manchester Royal Infirmary, Manchester, United Kingdom; 2 Division of Cellular Medicine, School of Medicine, University of St Andrews, St Andrews, United Kingdom; 3 Leeds Institute of Cardiovascular and Metabolic Medicine, School of Medicine, University of Leeds, Leeds, United Kingdom; 4 Division of Population and Behavioral Sciences, School of Medicine, University of St Andrews, St Andrews, United Kingdom; 5 Diabetes and Endocrinology Department, Somerset NHS Foundation Trust, Musgrove Park Hospital, Taunton, Somerset, United Kingdom

**Keywords:** coagulation, fibrin, gastric bypass, obesity, thrombosis

## Abstract

**Background:**

Obesity is a complex condition associated with prothrombotic fibrin networks that are resistant to fibrinolysis. Altered fibrin clot properties enhance cardiovascular risk and associate with a poorer prognosis following acute ischemic events. Bariatric surgery is commonly employed to improve cardiometabolic outcomes in individuals with obesity. However, the effects of this surgical intervention on fibrin clot properties have not been comprehensively studied.

**Objectives:**

To examine fibrin clot and lysis parameters in Roux-en-Y gastric bypass (RYGB) patients before and after surgery.

**Methods:**

The fibrin clot properties of 32 individuals living with obesity before and 9 months after RYGB surgery were determined using turbidimetric analysis. Correlation and regression analyses were used to identify relationships between clot properties and anthropomorphic and clinical measures.

**Results:**

RYGB surgery resulted in a significant reduction in adiposity-associated anthropometric measures as well as improvements in glycemia and lipid profile. Clot maximum absorbance was reduced from 0.43 ± 0.11 at baseline to 0.29 ± 0.10 at 9 months postsurgery (*P* < .0001), while fibrin clot lysis time failed to show a difference. The change in maximum absorbance was not caused by alterations in fibrinogen levels, while plasminogen activator inhibitor-1 concentration was significantly increased after surgery from 10,560 ± 6681 pg/mL to 15,290 ± 6559 pg/mL (*P* = .009). Correlation and regression analyses indicated that maximum absorbance was influenced by markers of adiposity as well as glycated hemoglobin and high-sensitivity C-reactive protein concentrations.

**Conclusion:**

RYGB surgery led to a decrease in the maximum absorbance of the fibrin clot. Values of maximum absorbance were associated with measures of glycemic control and inflammation. In contrast to previous reports, fibrin clot lysis time was not affected after surgery.

## Essentials

• Obesity is associated with a prothrombotic phenotype.
• Fibrin clot parameters were measured in patients before and after gastric bypass surgery.
• Fibrin clot maximum absorbance reduced after surgery, but lysis time did not change.
• Maximum absorbance was significantly related to measures of adiposity, glycated haemoglobin, and high-sensitivity C-reactive protein.

## 1 Introduction

Obesity associates with an enhanced cardiovascular risk [1] and is an independent risk factor for acute myocardial infarction [2], ischemic stroke [3], deep vein thrombosis, and pulmonary embolism [4]. Notably, changes in the plasma composition and/or activity of coagulation factors contribute to a hypercoagulable state in individuals living with obesity (ILWO). For instance, both tissue factor and factor VII are elevated in ILWO, leading to greater rates of thrombin generation [5]. Some anticoagulation mechanisms are also affected, likely due to impaired vascular endothelium functioning [6]. The abundance of clotting factors may also influence fibrin clot architecture and fibrinolytic efficiency. For instance, the levels of plasminogen activator inhibitor-1 (PAI-1) are increased in ILWO, which may lead to a suppression of fibrinolysis in this disease [7]. Moreover, circulating fibrinogen concentration is a major determinant of fibrin clot characteristics, and increased fibrinogen levels are commonly observed in ILWO [8,9]. Overall, diminished fibrinolysis and altered fibrin clot characteristics favoring the formation of dense clots that are more resistant to the fibrinolytic mechanism (hypofibrinolysis) may limit clot lysis in ILWO and thus contribute to increased persistence and severity of thrombotic events [10,11]. Importantly, derangements in coagulation and fibrinolysis in obesity may be exacerbated by comorbid conditions such as insulin resistance, hyperglycemia, and atheromatous dyslipidemia, which have been shown to influence fibrin clot characteristics [12,13].

Roux-en-Y gastric bypass (RYGB) surgery is commonly employed to induce weight loss and improve cardiometabolic outcomes in patients living with obesity. The surgery is associated with a reduction in all-cause and cardiovascular mortality as well as a lowered incidence of heart failure, coronary artery disease/myocardial infarction, and stroke [14]. The procedure can lead to a remission of type 2 diabetes and a restoration of lipid metabolism, which can occur rapidly and independently of weight loss [15,16]. Bariatric surgery associates with favorable changes in the hemostatic mechanism. These include reductions in tissue factor and FVIII concentrations and thrombin generation potential [17]. The surgery also associates with reductions in plasma fibrinogen and PAI-1 concentrations, which may lead to favorable changes in clot structure and fibrinolytic activity [18]. Notably, only two other studies have investigated *in vitro* fibrin clot structure and lysis after bariatric surgery. These studies showed that RYGB led to an improvement in clot lysis 6, 12, and 24 months after surgery [19,20]. A later substudy showed that the enhanced clot lysis 6 months after surgery was predicted by reductions in circulating fibrinogen and plasmin inhibitor (PI) [21]. It is noted, however, that the factors influencing fibrin clot structure and fibrinolysis are complex. Ultimately, more studies are needed to fully characterize the changes in fibrin clot properties that occur following surgery and to identify potential mechanisms that lead to such changes. Here, we have employed a validated turbidimetric assay to measure changes in fibrin clot properties in plasma samples taken from 32 individuals with obesity before and 9 months after RYGB. Moreover, fibrin clot parameters were investigated for relationships with anthropometric and clinical measures collected from the cohort before and 9 months after surgery.

## 2 Methods

### 2.1 Ethics statement

Blood was collected following approval by the National Research Ethics Service Committee Yorkshire & The Humber-Sheffield (Research Ethics Committee reference: 11/H1308/16) after obtaining written informed consent from all participants. All research protocols were performed in accordance with the Declaration of Helsinki.

### 2.2 Sample collection and treatment

Briefly, a total of 32 Roux-en-Y surgical patients (22 females and 10 males) were recruited from York Hospital, York, United Kingdom. Male or female participants over 18 years of age who were referred to the hospital for bariatric surgery for obesity were included in this study. Participants were excluded if they were <18 years old, diagnosed with an endocrine disorder other than type 2 diabetes, had a history of alcohol/drug abuse, had a significant psychological history, had a history of deep vein thrombosis, were taking any type of anticoagulant medication, were pregnant, had a history of active malignancy, or developed postoperative complications. With the exception of 1 participant (who was of Asian-British descent), all participants were Caucasians. Two weeks before surgery, patients were placed on a low-calorie diet (800-1000 kcal/d) and commenced taking multivitamin/mineral supplements. After surgery, patients were given a puree diet for 4 weeks and, after this time, advised to continue taking a multivitamin/mineral supplement. Blood was collected prior to surgery (within 48 hours of the procedure) and at 9-month follow-up following surgery. Blood was collected in either lithium heparin, EDTA, or citrated collection tubes after patients had fasted for at least 6 hours. Downstream laboratory investigations, including blood component analysis and assessment of *in vitro* fibrin clot properties, were performed using either EDTA-treated whole blood or plasma isolated from the lithium heparin or citrate-treated samples. Plasma was isolated within 30 minutes of blood collection. Plasma isolation involved centrifugation at 2400 × g for 20 minutes at 4 °C to remove cellular contaminants. Supernatant plasma was then aliquoted and stored at −80 °C until analysis.

### 2.3 Measurement of anthropometric characteristics

Anthropometric measurements were generated as expressed in the National Health and Nutrition Examination Survey III (Centers for Disease Control and Prevention). Body measurements were performed using a BC420SMA Body Composition Analyzer (Tanita).

### 2.4 Measurement of metabolic variables

EDTA whole blood was used to measure glycated hemoglobin (HbA1c) by the Laboratory Medicine Service at York Hospital using boronate affinity chromatography [22]. HbA1c concentrations are expressed as a percentage of total hemoglobin. HbA1c concentrations of  <6.0% (<42 mmol/mol) were considered normoglycemic as per WHO criteria. Cholesterol, triglycerides, high-density lipoprotein, and low-density lipoprotein cholesterol concentrations were measured in plasma collected in lithium heparin tubes, also by the Laboratory Medicine service at York Hospital, using standard methods.

### 2.5 Measurement of inflammatory and thrombotic markers

Total high-sensitivity C-reactive protein (hs-CRP) levels in citrated plasma were measured by the Leeds Pathology Blood Sciences service (Leeds). A Human PAI-1 ELISA Kit (Abcam) was used to measure PAI-1 in citrated plasma. Fibrinogen was measured using the Clauss method on an Amelung KC10 coagulometer (LABCON).

### 2.6 Assessment of fibrin clot properties

Fibrin clot properties were determined from citrated plasma using a validated turbidimetric assay [23,24]. Briefly, 10 μL of human thrombin at 200 U/mL (Calbiochem) was added to 90 μL of permeation buffer (50 mM Tris, 140 mM NaCl, pH 7.4). An activation mixture was then prepared by adding 54 μL of the diluted thrombin to a prepared mixture of 270 μL of 1 M CaCl_2_ and 11.7 mL of permeation buffer. A lysis mixture was prepared by adding 40 μL of 100 μg/mL tissue plasminogen activator (tPA) (Technoclone) to 760 μL of permeation buffer and then adding 500 μL of diluted tPA to 14.5 mL of permeation buffer. Citrated plasma samples (25 μL) were loaded into a 96-well microplate in duplicate, and 75 μL of the lysis mixture was added. After 3 minutes, 50 μL of the activation mixture was added. Therefore, the final concentration of each component was as follows: patient plasma diluted 1:6, 0.03 U/mL thrombin, 7.7 mM CaCl_2_, and 0.35 μg/mL tPA. After addition of the activation mixture, the microplate was immediately transferred to an ELx808 microplate reader (BioTek), and A_340_ was read at 12-second intervals for at least 2 hours at 37 °C using KC4 v2.7 software (BioTek).

The following parameters were determined from the resulting plasma turbidity curve: the lag time was taken as the time point at which absorbance rose by 0.01 above the baseline; the maximum absorbance was taken as the highest absorbance value recorded on the turbidity curve; the time to 50% lysis was taken as the time from the maximum absorbance to the time at which a 50% reduction in absorbance occurred; the 100% lysis time was taken as the time from maximum absorbance to the time at which absorbance values return to baseline; and the clot lysis area was taken as the area under the curve from the time at which the maximum absorbance was reached to the time at which the absorbance returned to baseline.

### 2.7 Statistical analysis and representation

Data are presented as mean ± SD. Graphs were generated, and statistical analysis was performed using Prism 9.0 (GraphPad Software) and R version 4 [25,26]. All variables before and after surgery were tested for normality using the Shapiro-Wilk test. Differences between groups were assessed using paired Student’s *t*-tests or Wilcoxon matched-pairs signed rank tests for normally and nonnormally distributed data, respectively. For dichotomous variables (dysglycemia, metformin use, and statin use based on the counts), significance was assessed using the chi-squared test. Correlations between linear variants were calculated using the repeated measures correlation as described previously [27,28]. The repeated measures correlation uses a modified analysis of covariance model to account for dependence introduced by multiple measurements on each individual. Univariate regression was also employed to further study the relationships between linear variables. To adjust for dependence introduced by the repeated measurements, regression analyses were performed using linear mixed models with random intercepts for individuals [29,30]. The significance threshold in all cases was *P* ≤ .05.

## 3 Results and Discussion

### 3.1 Patient recruitment and attrition

**Figure 1:**
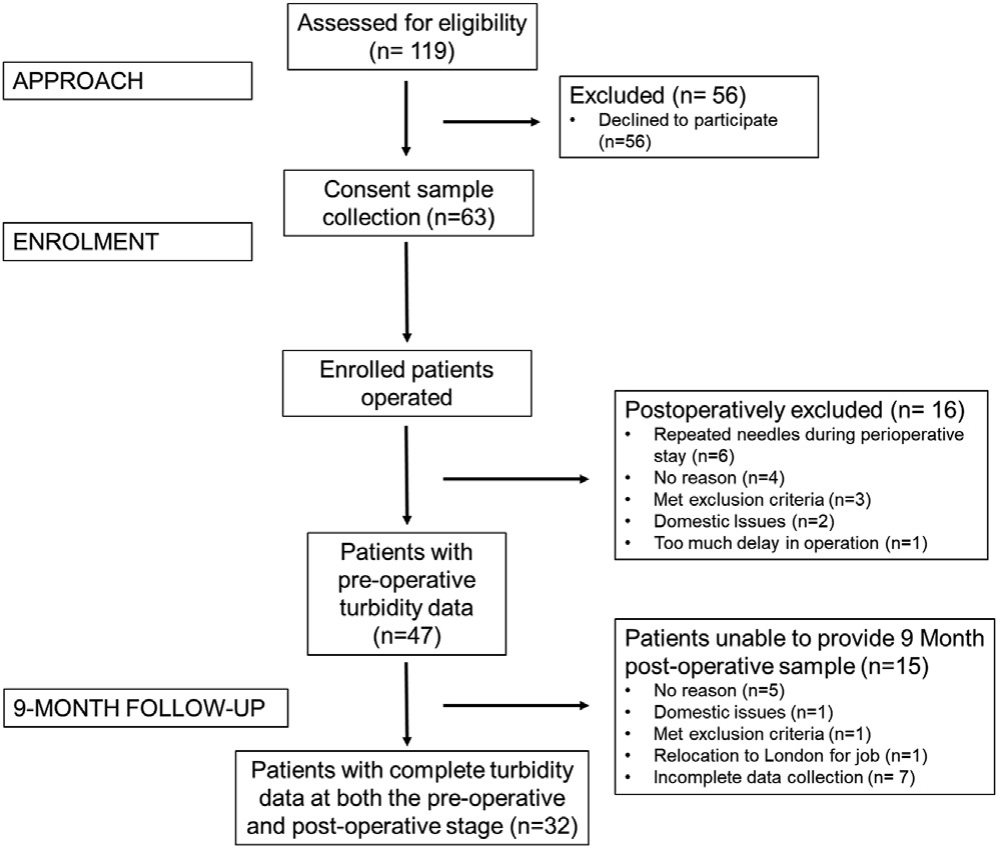
Flowchart showing patient recruitment and attrition.

As shown in the recruitment and attrition chart (Figure 1), 119 patients were approached for study participation. A total of 63 patients (42 females and 21 males) were recruited and consented (recruitment rate of 52.94%). Of the 63 patients, 16 were postoperatively excluded (attrition rate of 25%). Of the remaining 47 patients (31 females and 16 males), 15 failed to provide a valid 9-month follow-up sample (attrition rate of 32%). Overall, this study included 32 participants with complete blood component and turbidity data during both the pre- and postoperative period (22 females and 10 males).

### 3.2 Changes in anthropometric and clinical characteristics after surgery

Anthropometric values (including weight, body mass index, waist/hip/neck circumferences, and waist-to-hip ratio) and whole blood concentrations of HbA1c, glucose, and blood lipid profile were measured in the participants before and 9 months after surgery (Table 1). Overall, RYGB surgery led to significant reductions in all measured anthropometric markers, indicating that it was successful in reducing adiposity (including visceral adiposity). There were improvements in glycemic status, as indicated by reductions in glucose and HbA1c concentrations and number of patients requiring metformin. There was also evidence of improvement in lipid markers, including significant reductions in total cholesterol, triglycerides, low-density lipoprotein, cholesterol:high-density lipoprotein ratio, and number of patients requiring a statin.

**Table 1:** Demographic characteristics of the studied population and clinical characteristics, including mean anthropometric values, whole blood HbA1c, blood lipid profile, and prescribed medications before and 9 months after surgery (*N* = 32).

Characteristic	Values		
Age at recruitment (y) ± SD	47.91 ± 10.3		
Males % (*n/N*)	31.25 (10/32)		
Smokers % (*n/N*)	50.0 (16/32)		
	Presurgery	9 months postsurgery	*P* value
Weight (kg) ± SD	148.3 ± 24.9	105.0 ± 18.9	<.0001
BMI (kg/m^2^) ± SD	52.1 ± 7.3	37.5 ± 4.9	<.0001
Waist circumference (cm) ± SD	143.0 ± 10.4	116.7 ± 11.0	<.0001
Hip circumference (cm) ± SD	136.9 ± 11.5	117.9 ± 10.5	<.0001
Waist-to-hip ratio ± SD	1.1 ± 0.1	1.0 ± 0.1	<.0001
Neck circumference (cm) ± SD	46.3 ± 5.1	40.0 ± 4.6	<.0001
Excess weight (kg) ± SD	87.3 ± 22.0	44.2 ± 15.1	<.0001
Visceral fat rating ± SD	23 ± 7	14 ± 4	<.0001
Glycated hemoglobin (%) ± SD	6.9 ± 1.7	5.6 ± 0.7	<.0001
Glucose (mmol/L) ± SD	5.5 ± 2.5	4.6 ± 0.7	.014
With dysglycemia % (*n/N*)	62.5 (20/32)	15.6 (5/32)	<.0010
Prescribed metformin % (*n/N*)	31.25 (10/32)	12.5 (4/32)	.13
Total cholesterol (mM) ± SD	5.0 ± 1.1	4.5 ± 0.9	.0055
Triglyceride (mM) ± SD	1.9 ± 1.2	1.6 ± 1.0	.04
High-density lipoprotein (mM) ± SD	1.1 ± 0.2	1.2 ± 0.2	.08
Low-density lipoprotein (mM) ± SD	3.1 ± 1.0	2.8 ± 0.7	.04
Cholesterol:high-density lipoprotein ratio ± SD	4.7 ± 1.1	4.0 ± 0.9	.0002
Prescribed statin % (*n/N*)	31.25 (10/32)	18.75 (6/32)	.4

The *P* values were calculated using paired Student’s *t*-tests or Wilcoxon matched-pairs signed rank tests for normally and nonnormally distributed data, respectively. For dichotomous variables (dysglycemia, metformin use, and statin use based on the counts), significance was assessed using the chi-squared test.

BMI, body mass index.

### 3.3 Change in fibrin clot formation and lysis properties after surgery

**Figure 2:**
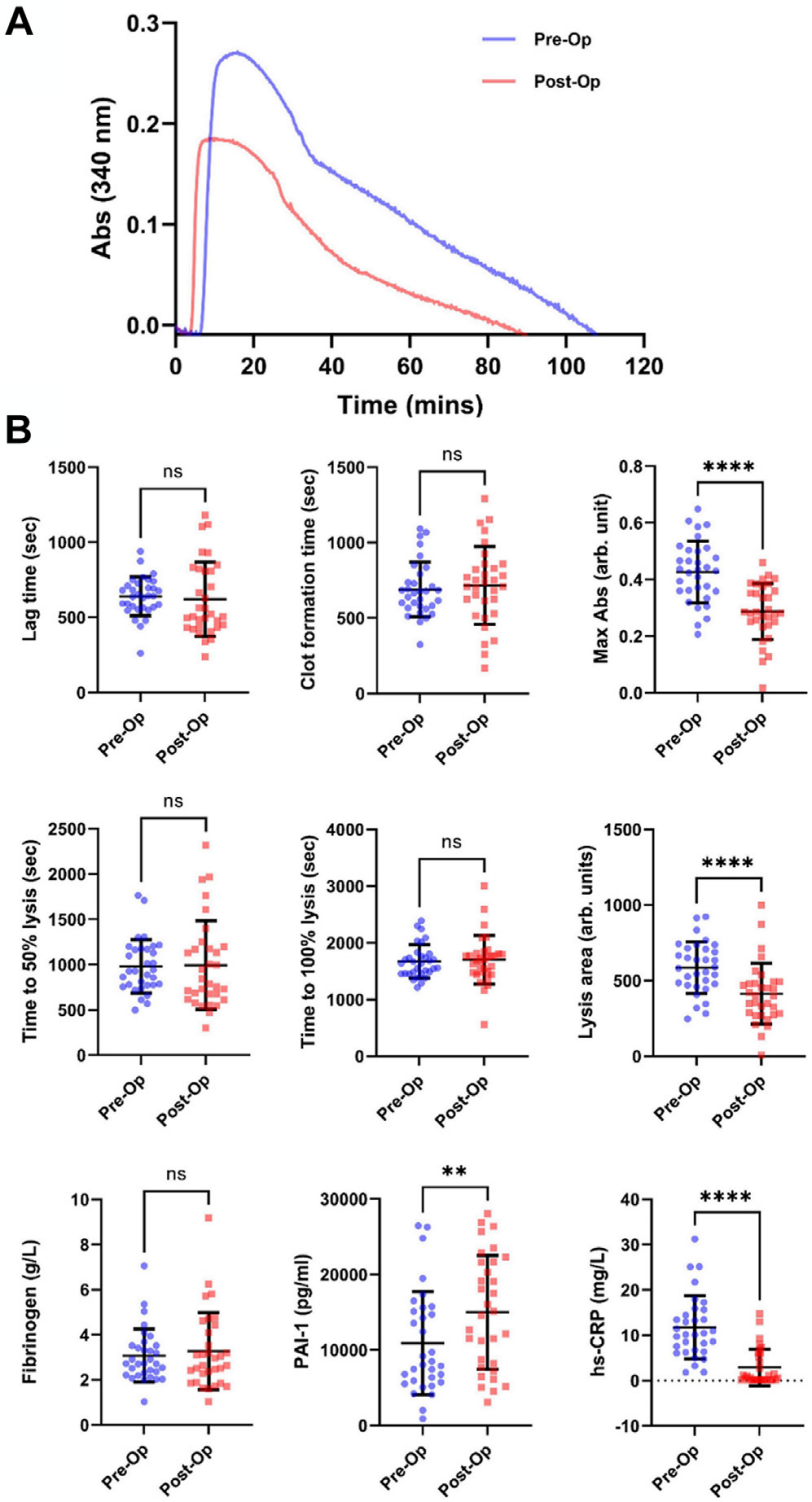
(A) Representative turbidity curves demonstrating fibrin clot formation and lysis generated from patient plasma before (blue) and 9 months after (red) Roux-en-Y gastric bypass surgery. Plasma clotting was initiated via the addition of thrombin and CaCl_2_ (final concentrations of 0.03 U/mL and 7.7 mM, respectively). Fibrinolysis was initiated via the addition of tissue plasminogen activator (tPA) (final concentration of 0.35 μg/mL). The formation and lysis of the resulting clot were tracked by monitoring absorbance (Abs) at 340 nm. Absorbance at 340 nm was monitored every 12 seconds for at least 2 hours at 37 °C. (B) Fibrin clot properties and fibrinogen, plasminogen activator inhibitor-1, and high-sensitivity C-reactive protein (hs-CRP) concentrations in patients living with obesity before (Pre-Op) and 9 months after (Post-Op) Roux-en-Y gastric bypass surgery (*N* = 32). Maximum absorbance and lysis area were measured in arbitrary (arb.) units. Error bars show the mean values ± SD. The *P* values were calculated using paired Student’s *t*-tests or Wilcoxon matched-pairs signed rank tests for normally and nonnormally distributed data, respectively. Statistical significance is indicated as ns; not significant (*P* > .05), ∗∗; *P* ≤ .01, and ∗∗∗∗; *P* ≤ .0001.

Clot formation and lysis properties were analyzed using a validated turbidimetric assay [23,24]. A representative turbidity curve before and after surgery is shown in Figure 2A. Fibrin clot parameters (including lag time, clot maximum absorbance, 50% lysis time, 100% lysis time, and lysis area) and the concentrations of fibrinogen, PAI-1, and hs-CRP were determined in all subjects before and 9 months after surgery (Figure 2B). The mean values of clot lag time, clot formation time, and lysis time did not significantly change after surgery. However, there was a decrease in both maximum absorbance (0.43 ± 0.11 arb. units to 0.29 ± 0.10 arb. units; *P* < .0001) and clot lysis area (587 ± 171 arb. units to 413 ± 201 arb. units; *P* < .0001). It is likely that the change in clot lysis area was mediated by the altered maximum absorbance, given the failure to detect a difference in lysis time and the absence of a difference in lysis area after adjusting for maximum absorbance. Overall, there was a reduction in maximum absorbance in the majority (84%) of participants following surgery. This change appears to be independent of fibrinogen concentration, which was not significantly altered after surgery. Association studies performed by Pieters et al. [31] have indicated that values of maximum absorbance determined by plasma turbidity assay are related to the overall density of the clot (volume occupied by fibers per total clot volume). Thus, it is conceivable that the reduction in maximum absorbance after surgery represents a structural change in the fibrin clot. To the best of our knowledge, this is the first study to show a change in maximum absorbance in response to bariatric surgery. It is noted that a concomitant reduction in maximum absorbance following clinical improvements in adiposity, glycemic status, and lipid metabolism in our cohort complements previous work by Carter et al. [12] who showed that values of maximum absorbance increased progressively with the number of metabolic syndrome components. Moreover, it is known that a higher maximum absorbance associates with increased cardiovascular risk in various patient groups [12,32,33]. Thus, the observed reduction in maximum absorbance after bariatric surgery may have clinical relevance.

Interestingly, plasma PAI-1 concentration increased following surgery (from 10,560 ± 6681 pg/mL to 15,290 ± 6559 pg/mL postsurgery, *P* = .01) in contrast to other studies (as reviewed in [18]). Plasma PAI-1 levels are typically elevated in individuals with obesity or metabolic syndrome [7]. This may be due to increased expression of PAI-1 by adipocytes in visceral fat stores [34]. Elevated PAI-1 in ILWO can be normalized through weight loss strategies, and bariatric surgery-induced reduction in visceral fat diameter has previously been shown to predict a decrease in PAI-1 levels [35,36]. Our findings may therefore represent a weight-independent mechanism of PAI-1 regulation warranting further investigation. It is also possible that the discontinuation of certain medications may contribute to the elevated PAI-1 postsurgery. Notably, both metformin and statin therapy are associated with reductions in PAI-1 [37,38]. Thus, it is possible that medicine discontinuation may contribute to the observed changes in PAI-1 after surgery. However, confounding effects of medication could not be meaningfully investigated in this study since only 5 out of 32 patients in our surgical cohort were medication-free.

Notably, clot lysis did not significantly change after surgery. This contrasts with previous studies by Stolberg et al. [19] and Dawson et al. [20], both of which showed a reduction in lysis time after surgery. Notably, a further substudy by Pedersen et al. [21] specifically linked a reduction in PI and fibrinogen 6 months after bariatric surgery to improved lysis. Therefore, it is conceivable that lysis times did not change due to the unaltered fibrinogen (and potentially PI) concentration. The smaller size of our study also may have hindered the ability to identify significant differences in lysis times after surgery. Notably, PAI-1 strongly influences *in vitro* clot lysis even when high concentrations of exogenous tPA are used in the assay [39]. Thus, it is possible that the postoperative increase in PAI-1 may have limited improvements in clot lysis. Finally, it is important to recognize that plasma clot lysis in both health and disease states has also been shown to be influenced by a range of both hemostatic and inflammatory proteins, including thrombin activatable fibrinolysis inhibitor, complement C3, and histidine-rich glycoprotein, which were not measured in this study [[39], [40], [41]]. Overall, these observations emphasize a need to consider a broader proteomic scope to fully explain the changes in plasma clot parameters that occur following RYGB surgery.

### 3.4 Relationships between maximum absorbance and clinical parameters

Maximum absorbance was investigated for relationships with anthropometric values and other clinical parameters. The correlations were computed using combined pre- and postoperative measurements (*N* = 64), with appropriate adjustments for dependence introduced by the repeated measures. The influence of each characteristic on maximum absorbance was also quantified using univariate regression (*N* = 64). To account for potential dependencies within the data due to repeated measurements, regression analysis was performed using a linear mixed model (LMM) with random intercepts for each individual. The results of the correlation and regression analyses are presented in Table 2.

**Table 2:** Results from correlation and regression analyses investigating the relationship between maximum absorbance with anthropometric and clinical measurements (*N* = 64).

Characteristic	Repeated measures correlation			Univariate regression (LMM)		
	Coefficient (*r*)	95% CI	*P* value	Beta	95% CI	*P* value
BMI	.693	0.53-0.83	<.0001	.006	0.003-0.01	<.001
Weight	.617	0.43-0.82	.0001	.002	0.001-0.003	<.001
Waist circumference	.717	0.56-0.84	<.0001	.004	0.002-0.01	<.001
Hip circumference	.622	0.48-0.75	.0001	.003	0.001-0.01	.004
Waist-to-hip ratio	.592	0.33-0.79	.0003	.451	0.12-0.78	.008
Neck circumference	.662	0.52-0.81	<.0001	.008	0.003-0.01	.002
Excess weight	.584	0.41-0.79	.0004	.002	0.001-0.003	<.001
Visceral fat rating	.75	0.61-0.87	<.0001	.009	0.006-0.01	<.001
Glycated hemoglobin	.443	0.22-0.81	.0099	.023	0.002-0.05	.04
High-sensitivity C-reactive protein	.575	0.34-0.78	.0005	.006	0.002-0.01	.006

Correlations were performed using the repeated measures correlation. Regression analysis was performed using a LMM with random intercepts for individuals. The regression output shows that the coefficient estimates (beta) are quite close to zero; most are below 0.01. This reflects the compact scale and small magnitude of maximum absorbance (minimum: 0.017, maximum: 0.6493).

BMI, body mass index; LLM, linear mixed model.

Maximum absorbance significantly correlated with all measured markers of adiposity: body mass index, weight, waist circumference, hip circumference, wait-to-hip ratio, neck circumference, excess weight, and visceral fat rating. Furthermore, regression analysis confirmed that adiposity measurements were significant predictors of maximum absorbance. This agrees with other reports that adiposity (particularly visceral adiposity) is a significant determinant of fibrin clot properties [12]. Building on this, our results demonstrate that bariatric surgery-induced weight loss leads to changes in fibrin clot characteristics in patients with obesity.

Maximum absorbance was significantly correlated with HbA1c concentration (*r* = .443; *P* = .0099). Based on the regression, each percent decrease in HbA1c is associated with an average decrease in max absorbance of 0.023 (*P* = .04). These findings are in agreement with several other groups that have also shown an ability of glycemic status to influence *ex vivo* plasma clot properties, including values of maximum absorbance [42,43]. HbA1c was reduced following surgery, and thus, normalization of obesity-associated dysglycemia may have led to the changes in maximum absorbance after surgery.

Maximum absorbance was also significantly correlated with hs-CRP (*r* = .575; *P* = .0005). Regression analysis indicates that each 1 mg/L decrease in hs-CRP corresponds to an average decrease in maximum absorbance of 0.006 (*P* = .006). These results agree with several other studies that indicate a role for CRP to regulate clot maximum absorbance [31,44]. Notably, surgery led to a significant reduction in CRP levels (from 11.73 ± 6.95 mg/L to 2.88 ± 4.04 mg/L postsurgery), indicating a broad decrease in systemic inflammation. Our results suggest that the remission of the inflammatory milieu may have led to the changes in maximum absorbance postsurgery.

In summary, RYGB surgery leads to substantive weight loss and a reduction in fibrin clot maximum absorbance. Correlation and regression analyses indicate that the decrease in maximum absorbance may be driven, in part, via surgery-induced improvements in glycemic status and/or the inflammatory state.
